# Quality of life and associated factors among women with obstetric fistula in Ethiopia

**DOI:** 10.1186/s12905-021-01458-3

**Published:** 2021-08-28

**Authors:** Biruktawit Matiwos, Getachew Tesfaw, Asmare Belete, Dessie Abebaw Angaw, Shegaye Shumet

**Affiliations:** 1 Ras Desta Hospital, Addis Ababa, Ethiopia; 2 Department of Psychiatry, University of Gondar, Gondar, Ethiopia; 3 Amanuel Mental Specialized Hospital, Addis Ababa, Ethiopia; 4 Department of Epidemiology and Biostatistics, University of Gondar, Gondar, Ethiopia

**Keywords:** Quality of life, Fistula, Ethiopia

## Abstract

**Background:**

Obstetric fistula is an abnormal opening between the vagina and bladder or rectum. Women affected by obstetric fistula are often abandoned by their husbands, stigmatized by the community, physically debilitated and blamed for their conditions. These factors lead the victims to low self esteem, depression and prolonged emotional trauma. The physical, emotional and social suffering associated with continuous leakage of urine has a profound impact on women quality of life. The aim of this study was to assess quality of life and associated factors among obstetric fistula patients in Ethiopia, and it will have a significant role for further intervention.

**Objective:**

To assess quality of life and associated factors among obstetric fistula patients in Ethiopia, 2017.

**Methods:**

Institution based cross-sectional study design was conducted at fistula centers in Ethiopia. Systematic sampling technique was used to recruit a total of 289 women with obstetric fistula. The World Health Organization Quality of Life—Brief (WHOQOL-BREF) Version was used to assess quality of life. We computed simple and multiple linear regression analysis to assess factors associated with quality of life and *P*-value < 0.05 was declared statistically significant. Adjusted unstandardized β coefficient of multiple linear regressions was used to describe associated factors of quality of life.

**Result:**

Of 289 women studied, only 12.1% felt satisfied with their general state of health and quality of life. In the physical health domain, the mean quality of life score was 40.78 ± .78. In the psychological domain, the mean quality of life score was 39.96 ± .82. In the social and environmental domain, the mean quality of life score was 32.9 ± .95, 36.45 ± .8, respectively. Duration of incontinence (β =  − 3.8,95% CI(− 6.95, − .62), patients coming for surgical procedure (β =  − 4.4, 95% CI(− 7.64, − 1.2), poor social support(β =  − 6.14, 95%CI (− 8.8, − 3.4), co-morbid anxiety (β =  − 4, 95% CI (− 7,-1.1) and depression (β =  − 9.2, 95% CI (− 12, − 6.4) were negatively associated with physical domain of quality of life. Co-morbid anxiety (β =  − 11,95% CI (− 14.8, − 7.3), employment (β = 9.1,95% CI (.5, 17.6), number of children(β = 2.1,95%CI(.8, 3.4), and depression(β =  − 6.3,95%CI(− 9.7, − 2.9) were associated with a psychological domain. Duration of incontinence (β =  − 8.1, 95%CI(− 12.82, − 3.4), poor social support (β =  − 7.8(− 12, − 3.6), patients coming for surgical procedure (β =  − 12, 95%CI (− 17.4, − 6.4) and co-morbid anxiety (β =  − 9.2, 95% CI (− 13.8, 4.5) were negatively associated with social domain of quality of life. Number of children present (β = 2.4, 95%CI (.82, 3.6), and poor social support (β =  − 5.5, 95%CI (− 9.5, − 1.5) were significantly associated with an environmental domain of quality of life.

**Conclusion and recommendation:**

Co-morbid depression and anxiety, poor social support, duration of urine incontinence, employment, number of children, and duration of hospital stay were factors significantly associated with domains of QOL. Treating co-morbid depression and anxiety, and social support are necessary to increase women’s quality of life. In addition, it is better to have a plane of income generation victims, and awareness creation about early treatment of the problem for community by the concerned body to improve women quality of life.

## Introduction

Obstetric fistula is an abnormal connection between the vagina and rectum or between the vagina and bladder due to different reasons, like product of prolonged, obstructed and unattended labor [1]. Globally, greater than two million women were living with obstetric fistula. In Ethiopia, 10.6 per 1000 women were living with this problem [2].

Severe birth injuries like obstetric fistula has a profound effect on women's quality of life [3].Quality of life has a broad ranges of concepts which include individual's perception of their position in life in the context of culture and value systems they live, and in relation to their goals, expectations, standards, and concerns [4]. In developing countries, women’s quality of life is affected by persistent leakage of urine which results in perinea wetness, excoriation and pain, and a pervasive urinary odor [5]. It is one of the three chronic health conditions that most adversely affect an individual’s health-related quality of life (HRQOL) [6]. Although it is not life-threatening, failure to control sphincters can severely affect a woman's quality of life by limiting their physical, social, psychological and sexual functioning [7, 8]. In a debilitating conditions, in whichever form, sweepingly affects the QOL of the patients through generating feelings of anger and sadness, embarrassment, loss of self-esteem. Many fistula cases occur among women in traditional cultures, where women's status and self-worth may depend almost entirely on marriage and childbearing [9]. Sexual activity may be restricted or avoided entirely due to the crippling effect of the disease [10]. Fistula patients do not only suffer from physical discomfort, but they may also have to get the deal with its far-reaching effect on their emotional, psychological well-being and sleep deprivation following this morbidity [11].

Coexistent of severe depression and other mental co-morbidities are common among fistula patients, which affects the patient perception about incontinence, and altering QOL and general functioning of the patients [12, 13].

Despite obstetric fistula is treatable, it still remains a profound stigma and feeling of humiliation attached to these condition even after surgical repair [13] and result in impaired quality of life. QOL impairment among fistula patients is an obstacle in good physical and social well-being and to the patient's maintenance of general health and treatment [14]. Understanding health related quality of life is considered as an essential outcome for clinical trials and management [15]. The goal of treating the whole bladder should be to not only repair the fistula but improve QOL as well [16]. In developing countries including Ethiopia because of low skilled obstetric care access and low infrastructure to reach health facility for labor, women frequently suffer from obstetric fistula. And this problem adversely affects women’s health-related quality of life. Therefore; the aims of this study was to assess quality of life and associated factors among obstetric fistula patients to prevent further complication and helps to policy makers as an input to improve quality of life of women.

## Methods

### Study setting and population

An institution based cross-sectional study design was carried out among fistula patients in Addis Ababa Hamlin fistula hospital and outreach centers in Ethiopia between May and July 2017. These fistula hospitals were built in five permanent outreach centers in strategic locations of Ethiopia in addition to the main hospital in Addis Ababa. These five hospitals include Bahirdar and Mekele in northern, Yirgalem in southern, Metu in southwestern and Harar in the eastern part of Ethiopia. Each outreach centers have a ward, an operating room, educational and administrative facilities, as well as other essential facilities. These outreach centers were built adjacent to existing regional hospitals and offer a discrete entrance for fistula patients and women at high risk of obstructed labor. The main hospital in Addis Ababa is dedicated to treat complicated cases like fistula related injuries and supporting other regional Hamlin Fistula Centers. The village Desta Mender in Addis Ababa is involved in teaching and training patients that cannot be fully cured in addition to giving care for them. After considering the average number of patients per month in each center, systematic random sampling technique was used to select the study participants from the outpatient and inpatient department. The study included patients aged 15 years and above during data collection in each institution. Patients seriously ill and unable to communicate were excluded.

### Measurements

Patients’ quality of life was assessed by 26 items of WHOQOL-BREF questionnaire. The questionnaire consists of 2 parts. The first part evaluates the individual’s overall perception of quality of life and the individual’s overall perception of their health. The second part evaluates the 4 domains: physical health, psychological health, social, and environment health domain. Domain scores are scaled in a positive direction (i.e. higher scores correspond to better quality of life). QOL raw scores are transformed into a range between 0 and 100. The overall quality of life was computed as the average of the score of the four domains. The higher mean score indicates better the quality of life and vice versa.

Social support was assessed by using the Oslo three-item social support scale. The sum score ranges from 3 to 14 with 3 categories: 3–8 = poor social support; 9–11 = moderate social support; and strong social support 12–14. Perceived stigma was assessed by using a three-item Jacoby perceived stigma scale. Each of the three items requires a simple yes/no response. Discrimination experience was assessed using daily discrimination scale with the cutoff point’s one and above. Anxiety and depression were assessed by using Hospital anxiety and depression scale (HADS) with the cutoff point eight and above. Anxiety and depression symptoms were measured using hospital Anxiety and Depression Scale (HADS). It has 14 items and two subscales. Anxiety subscale (HADS-A) and depression subscale (HADS-D). Patients who scored ≥ 8 for both anxiety and depression symptoms were considered as having depression and anxiety.

### Data collection

Data were collected by face-to-face interviews using a semi-structured questionnaire. The questionnaires include socio-demographic, quality of life, social support, anxiety and depression, perceived stigma, and discrimination experience. The questionnaire was designed in English and translated to Amharic, Aphan Oromo and Tigrigna and back to English to maintain consistency. We conducted a reliability analysis for the translated version of the tools and showed a high score (Cronbach α = 0.83).

Data were collected by trained data collectors using the translated version of the questionnaires for a month. Data collectors were trained on how to interview patients and explain unclear questions and the purpose of the study. Furthermore, they were made aware about ethical principles, such as confidentiality/ anonymity/ data management, and securing respondents’ informed consent for participation.

### Data processing and analysis

Data were checked for completeness and consistency and entered into Epi-Data software version 3.1 and then exported into SPSS version-20 for analysis. The frequency, percent, mean and standard deviation were used to summarize the distribution of variables. The simple linear regression and multiple linear regression analysis were implemented to test an association between quality of life and the independent variables. During simple linear regression analysis, variables with *p*-value less than 0.05 were selected for further analysis in multiple linear regression analysis. Adjusted unstandardized β coefficient was used to describe associated factors for quality life at 95% confidence interval. Statistical significant was declared at *p*-value less than 0.05.

### Ethical consideration

Ethical clearances were obtained from the Ethical Review Committees of the University of Gondar and Amanuel Mental Specialized Hospital. A formal letter of permission was obtained from Amanuel Mental Specialized hospital and submitted to Hamlin fistula Ethical Review Committees to get permission for conducting the study in each hospital. We received written informed consent from study participants and assent from their guardians after explain the purpose of the study. Confidentiality was maintained by omitting their personal identification.

## Results

A total of 289 participants took part in the study, with a response rate of 90%. The mean (SD) age of the participants was 27(6.13) years. Nearly, half of the patients, i.e. N = 148(51.2%) were lived with their spouse; 175(61%) were Orthodox Christian; 157(54%) were unable to read and write, and 144(50%) were jobless with their occupational attainment (Table 1).

**Table 1:** socio-demographic characteristics of women with obstetric fistula in Ethiopia, 2017(n = 289)

Variables	Categories	Frequency	Percent (%)
Age		27 ± 6.13	
Religions	Orthodox		61
	Muslim		19.8
	Protestant		19.2
Marital status	With spouse	148	51.2
	Without spouse	141	48.8
Education	Unable to read and write	157	54
	Primary school	121	42
	Secondary school and above	11	4
Occupation	Private business	132	45.5
	Jobless	144	50
	Others*	13	4.5

Others* = students + employed

### Obstetric and fistula related characteristics of the respondents

The majority of the respondents, 218(75.4%) had VVF; 71(24.6%) had RVF/ RVF + VVF types of fistula; 125(43.4%) of the patients were incontinent for a year and below a year without any treatment. More than half, i.e. N = 169(58.5%) had at least one child, and N = 160(55.4%) had history of still birth. The mean (SD) age of the participants at the first marriage, and first pregnancy was 15.65 ± 3.6, 17.49 ± 3.73 years, respectively (Table 2).

**Table 2:** Distribution of obstetric and fistula related factors among women with obstetric fistula in Ethiopia,2017 (n = 289)

Variables	Categories	Freq/mean ± SD	Percent (%)
Mean age at the first marriage		15.65(SD ± 3.6)	
Mean age at first pregnancy		17.49 ± 3.73	
Type of fistula	VVF	218	75.4
	RVF/ RVF + VVF	71	24.6
Duration of incontinence before surgical repair	< 1	125	43.4
	1–5	103	35.4
	> 5	61	21.2
Women who have children	Yes	169	58.5
	No	120	41.5
Number of children present	Mean (SD)	2 ± 1.32	
stillbirth	Yes	160	55.4
	No	129	44.6
Mean number of previous stillbirths	2 ± 1.22		
Reason for coming to the institution	New visit to seek help	45	15.6
	Waiting for surgical procedure	190	65.7
	For follow up	17	5.9
	For counseling	4	1.4
Intervention	Surgery	223	77.2
	Counseling	10	3.5
	physiotherapy	4	1.4
Duration for follow ups	< 4 years	197	68.2
	4–8 years	47	16.2
	> 8 years	45	15.6
Hospital stay for intervention	< 6 month	155	53.6
	1–6 month	109	37.7
	> 6 months	25	8.7

VVP = Vesico vaginal fistula, RVT = Recto Vaginal Fistula

### Psychosocial factors

Regarding psychosocial factors of respondents, 152(52.6%) had poor social support; 49(30.4%) had moderate social support; 240(83%) were stigmatized, and 243(84%) had experience of discrimination (Table 3).

**Table 3:** Psychosocial factors among women with obstetric fistula in Ethiopia, 2017 (n = 289)

Variables	Categories	frequency	Percent (%)
Social support	Poor	152	52.6
	Moderate	88	30.4
	Strong	49	17
Perceived stigma	Stigmatized	240	83
	Non stigmatized	49	17
Discrimination experience	Discriminated	243	84
	Non discriminated	46	16

### Prevalence of depression and anxiety among respondents

**Fig. 1:**
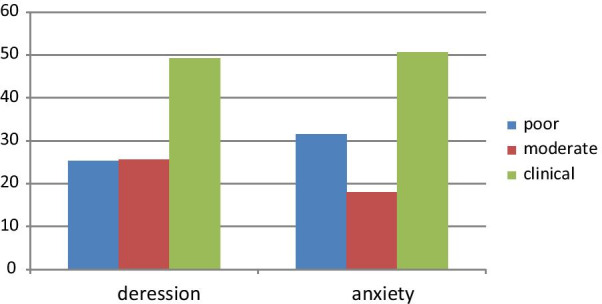
Prevalence of anxiety and depression among women with obstetric fistula in Ethiopia, 2017 (n = 289)

Concerning the co-morbid mental illness, 51.2% and 49.1% of the women had co-morbid anxiety and depressive symptoms, respectively (Fig. 1).

### Quality of life among obstetric fistula patients

In the current study, from the total participants 12.1% had felt satisfied for their general health status. The overall mean score of quality of life among fistula patients was 37.52 (95% CI 36.26, 38.73) with a standard deviation of 10.99. Mean score for each domain of WHOQOL were 40.78 ± 0.78 for physical domain; 39 0.96 ± 0.82 for psychological; 32 0.9 ± 0.95 for social domain, and 36 0.45 ± 0.8 for environmental domain of quality of life (Table 4).

**Table 4:** Quality of life among women with obstetric fistula in Ethiopia, 2017 (n = 289)

Domain	Mean score of QOL (SD)	Percentage of participants who score below the mean (%)	95%CI
Physical	40. 78 ± .78	52.2	(39.12, 42.31)
Psychological	39 .96 ± .82	27.3	(38.21, 41.43)
Social	32 .9 ± .95	34.9	(30.82, 34.89)
Environmental	36 .45 ± .8	53.6%	(34.84, 38.09)
Overall QOL	37.52 ± 10.99		(36.26,38.73)

### Factors associated with quality of life

Simple linear regression analysis was carried out between WHO-BREF domains and each independent variable. Variables with *p*-value ≤ 0.05 during simple linear regression analysis were selected for further analysis in multiple linear regression analysis. Age, educational status, age at first marriage, age at first pregnancy, type of fistula, incontinence, the reason why the women come to the institution, duration of hospital stay, social support, anxiety, depression, perceived stigma, discrimination were associated with physical domain. For psychological domain: age, educational status, occupation, age at first marriage, age at first pregnancy, type of fistula, incontinence, number of children, the presence of stillbirth, a reason why the women come to the institution, social support, anxiety, depression, perceived stigma, and discrimination were associated factors.

For social domain: age, occupation, urine incontinence, number of children, a reason why the women come to the institution, social support, depression, perceived stigma were factors associated with social domain of quality of life.

For environmental domain: marital status, educational status, presence of children, number of children, reason why the women come to the institution, duration of hospital stay, social support, anxiety, depression, perceived stigma, discrimination were factors associated with environmental domain of quality of life.

Results of multiple linear regressions showed that: women incontinent for more than 5 years, women with co-morbid anxiety and depression were significantly associated with physical domain. Four explanatory variables in the psychological domain i.e. women's' who have been employed in private, number of children, co-morbid anxiety and depression were associated factors. Urine incontinence, poor social support, women come to surgery and anxiety were factors associated with social domain, and number of children and social support were also factors statistically significant with environmental domain of quality of life (Table 5).

**Table 5:** Factors associated with quality of life domains among women with obstetric fistula at Addis Ababa fistula hospital and outreach centers, Ethiopia, 2017 (n = 289)

variables	Quality of life domain											
	physical			Psychological			Social			Environmental		
	Crude unstβ(95%CI)	Adj unst β (95%CI)	R^2^	Crude unstβ(95%CI)	Adj unst β (95%CI)	R^2^	crude unstβ(95%CI)	Adj unst β (95%CI)	R^2^	Crude unstβ(95%CI)	Adj unst β(95%CI)	R^2^
Duration of Incontinence	− 0.21(− 0.67, − 0.04	− 3.8(− 6.95, − 0.62) **	0.04	–	–	–	0.20 (2.18,6.74	− 8.1(− 12.82, − 3.4) ***	0.04		–	–
Patients visit for surgery	− 0.25(− 0.55, − 0.12	− 4.4(− 7.64, − 1.2) **	0.05	–	–	–	− 0.33(− 2.70, − 0.28	− 12(− 17.4, − 6.4) ***	0.11	–	–	–
social support	0.48(0.10,053)	− 6.14(− 8.8, − 3.4) ***	0.23	–	–		0.48(0.01,2.17)	− 7.8(− 12, − 3.6) ***	0.23	0.44(0.05,8.14)	− 5.5(− 9.5, − 1.5) ***	0.20
Anxiety	− 0.50(− 0.32, − 0.10)	− 4(− 7, − 1.1) **	0.26	− 0.62(− 7.21, − 3.48	− 11(− 14.8, − 7.3) ***	0.38	− 0.43(− 6.41, − 2.80)	− 9.2(− 13.8,4.5) ***	0.19	–	–	–
Depression	− 0.57(− 0.25, − 0.01	− 9.2(− 12, − 6.4) ***	0.33	− 0.50(− 1.72, − 0.08	− 6.3(− 9.7, − 2.9) ***	0.25	–	–	–	–	–	–
private employed	–	–	–		9.1(.5,17.6), **	0.02	–	–	–	–	–	–
Number of children present	–	–	–		2.1(0.8,3.4) **	0.14	–	–	–	0.12(0.04,2.15)	2.4(0.82,3.6) ***	0.07

^**^ stands for *p* < .005, ***stands for *p* < .001, (–) factors has no association, Unst.β = unstandard β, R^2^ = model fitness

## Discussion

In Ethiopia, like in other remote areas of poor countries, most women who develop fistula give birth at home without assistance from skilled attendants. These women also face related complications like physical or psychosocial issues which result in compromised quality of life. In this study, women’s mean quality of life in physical, psychological, social and environmental domains of QOL were 40.78 ± .776, 32.90 ± .952, 39.96 ± .817 and 36.45 ± .802, respectively. This low quality of life of women might be related with their intense fear of developing another fistula, which is commonly aggravated by sexual intercourse or giving birth. This fear might result in isolation, marital conflict, and/or economic vulnerability. Some women also continue to experience mental anguish, stigma and physical problems regardless of the success of the procedure [4]. This finding is supported by other studies result [17–19]. For example, results of a study conducted in western Ethiopia showed 35% of women experienced stress and urge incontinence after surgical procedure and had poor social reintegration. Another study in Nigeria also proved that unsuccessful repairs of the defects could result in low quality of life.

Regarding predictor variables, women with urine incontinent for greater than five years had decreased in their physical and psychological domain of quality of life. Women with long duration of incontinence might have unable to control leakage, poor sleep satisfaction and low daily activity, and negative feeling about themselves, which finally result in depression. Study showed 14% of women attempted suicide because of depressive symptoms [17].The finding is consistent with other studies conducted in Ethiopia and India [17, 18, 20]. Repairing the defects with surgery could significantly increase the physical and psychosocial quality of life of women [17]. In this study, women who were coming to the health institutions by appointment for surgical procedure were decrease in their physical quality of life by 4 in every unit increase in their appointment time for the procedure. The emotion related to incontinence might dissuade them to travel by public transport because majority of fistula cases come from distance area. For example; a study in rural west Ethiopia [17] showed that participants reported people used to hold their nose, laugh or talk about the smell when they approached. The other possible reason might be fear to die by complications secondary to surgical procedure, which induces stress and some physical symptoms. Women with multiple visits at the health institutions for surgical procedure had low social quality of life. This might be due to fear of lack of social assistance at hospital because of frequent appointment and visit, and women with continues leakage of urine have public stigma during transportation and other public gathering areas.

Poor social support was negatively associated with physical, social and environmental domain of QOL. Women with physical challenges might need assistance from husband, family members, relatives and community members; but a study has shown that women with fistula are more frequently divorced as result of this condition [21]. The lack of or poor social assistance after surgical procedures, might lead them to anger and sadness [22, 23]. The emotion related to physical challenges might also affect their relationships at home, with friends and husband. These feelings result in a loss of control over daily routines i.e. self care, household chores, and fulfill the traditional role of wife in the community [22]. Because of stigma, many women were not open to advocate for their fistula care and relationship needs, which brings low linkages to income generation opportunities [4, 16]. This is supported by studies in Malawi and Tanzania [24, 25].

Due to different reasons anxiety and depressive symptoms are common among women with obstetric fistula. For example, results of studies [26, 27] showed between 23.3 and 38.8% of women with fistula had major depressive symptoms and about half of the patients have co-morbid anxiety. The occurrences of anxiety and depressive symptoms might be related with women’s loss of child, loss of ability to work and acceptance, lack of social support. For example, in the current study, we found that the mental health of the women was increased by 2 in case of women who had children still alive. In this study, the presence of anxiety symptoms affected patients’ physical, psychological and social quality of life, what might be related to stigma. The stigmatization of anxiety might cause a woman to have diminished self worth and confidence, and socially disconnected from social activity and spend their time alone spent [22]. This finding is consistent with other study findings [27, 28].

Women with obstetric fistula who were employed had good psychological quality of life i.e. the mental health quality of life of women was increase by 9 when they were employed. This might be due to Employment increases mental health status of women and reduces the long term mental health care expenditure of women with mental health disabilities. A study showed 50% of women with fistula economically impoverished by job loss [21]. When people lose their jobs, they tend to experience a significant deterioration in mental health. In most rural area of Ethiopia, women are powerless in their married lives; so women with fistula stop working and remain at home [22], what make them even more dependent of their family, and adversely affect their mental health condition.

### Limitations of the study

This study has several limitations. Social desirability and recall bias might be present. Because of data collection methods were face-to-face interview, which might lead individuals to respond socially acceptable answers during the interview process. The translated tool, WHOQOL, was not validated in this study although it was widely used to measure quality of life in Ethiopia.

The design of the study was cross-sectional; therefore, we were unable to establish causal relationships from the observed results.

This study was a quantitative approach by using different instruments or scales, but further studies will benefit from qualitative or mixed (quantitative plus qualitative) study designs.

Furthermore, we did not assess patients’ change of quality of life before repair and after repair of obstetric fistula.

## Conclusion

In this study, quality of life among obstetric fistula patients was low. Depression, anxiety, poor social support, incontinence, employment, number of children present were factors associated with quality of life. Therefore, we recommend clinicians to consider co-morbid depression and anxiety, social support and income generation opportunities to increase quality of life.

We also suggest that Ministry of Health promotes community awareness about early treatment of obstetric fistula. Moreover; the government may develop specific programs for the victims, improving their quality of life through job generation and stable income.

## Data Availability

All the data are included in the manuscript. If anyone who needs extradata can access from primary author (birktawitm@gmail.com).

## Abbreviations

HRQOL: Health-related quality of life
OF: Obstetric fistula
QOL: Quality of life
RVF: Recto vaginal fistula
UI: Urinary incontinence
UoG: University of Gondar
VVF: Vesico-vaginal fistula
WHO: World Health Organization
WHOQOL: World health organization quality of life

## References

[1] Wall LLJTL (2006). Obstetric vesicovaginal fistula as an international public-health problem. Lancet.

[2] Vangeenderhuysen C, Prual A, Joud DO (2001). Obstetric fistulae: incidence estimates for sub-Saharan Africa. Int J Gynecol Obstet.

[3] Schultz SE, Kopec JA (2003). Impact of chronic conditions. Health Rep.

[4] Donnelly K, Oliveras E, Tilahun Y, Belachew M, Asnake MJ (2015). Quality of life of Ethiopian women after fistula repair: implications on rehabilitation and social reintegration policy and programming. Am J Public Health.

[5] Berlim MT, Fleck MJ (2003). “Quality of life": a brand new concept for research and practice in psychiatry. Braz J Psychiatry.

[6] Science WGJS, medicine. The World Health Organization quality of life assessment (WHOQOL): position paper from the World Health Organization. 1995;41(10):1403–9. 10.1016/0277-9536(95)00112-k 8560308

[7] Herzog AR, Diokno AC, Brown MB, Fultz NH, Goldstein NE (1994). Urinary incontinence as a risk factor for mortality. J Am Geriatr Soc.

[8] Walker GJ, Gunasekera PJ (2011). Pelvic organ prolapse and incontinence in developing countries: review of prevalence and risk factors. Int Urogynecol J.

[9] Mselle LT, Moland KM, Evjen-Olsen B, Mvungi A, Kohi TWJ (2011). "I am nothing": experiences of loss among women suffering from severe birth injuries in Tanzania. BMC Womens Health.

[10] Vigod SN, Stewart DE (2006). Major depression in female urinary incontinence. Psychosomatics.

[11] Kabir M, Iliyasu Z, Abubakar I, Umar UJ (2003). Medico-social problems of patients with vesico-vaginal fistula in Murtala Mohammed Specialist Hospital, Kano. Ann Afr Med.

[12] Zeleke BM, Ayele TA, Woldetsadik MA, Bisetegn TA, Adane AA (2013). Depression among women with obstetric fistula, and pelvic organ prolapse in northwest Ethiopia. BMC Psychiatry.

[13] Melville JL, Walker E, Katon W, Lentz G, Miller J, Fenner DJ (2002). Prevalence of comorbid psychiatric illness and its impact on symptom perception, quality of life, and functional status in women with urinary incontinence. Am J Obst Gynecol.

[14] Rowles SV, Prieto L, Badia X, Shalet SM, Webb SM, Trainer PJ (2005). Quality of life (QOL) in patients with acromegaly is severely impaired: use of a novel measure of QOL: acromegaly quality of life questionnaire. J Clin Endocrinol Metab.

[15] Feeny D, Furlong W, Saigal S (2004). Sun JJ Comparing directly measured standard gamble scores to HUI2 and HUI3 utility scores: group-and individual-level comparisons. Soc Sci Med.

[16] De Bernis LJ (2007). Obstetric fistula: guiding principles for clinical management and programme development, a new WHO guideline. Int J Gynecol Obstetr.

[17] Nielsen H, Lindberg L, Nygaard U, Aytenfisu H, Johnston O, Sørensen B (2009). A community-based long-term follow up of women undergoing obstetric fistula repair in rural Ethiopia. BJOG Int J Obstetr Gynaecol.

[18] Singh V, Jhanwar A, Mehrotra S, Paul S, Sinha RJ (2015). A comparison of quality of life before and after successful repair of genitourinary fistula: Is there improvement across all the domains of WHOQOL-BREF questionnaire?. Afr J Urol.

[19] Umoiyoho A, Inyang-Etoh E, Abah G, Abasiattai A, Akaiso O. Quality of life following successful repair of vesicovaginal fistula in Nigeria. 2011;11(3). 21905761

[20] Browning A, Menber BJ (2008). Women with obstetric fistula in Ethiopia: a 6-month follow up after surgical treatment. BJOG Int J Obstetr Gynaecol.

[21] Gharoro E, Agholor KJ (2009). Aspects of psychosocial problems of patients with vesico-vaginal fistula. BJOG Int J Obstetr Gynaecol.

[22] Gebresilase YT (2014). A qualitative study of the experience of obstetric fistula survivors in Addis Ababa, Ethiopia. Int J Women's Health.

[23] Imoto A, Matsuyama A, Ambauen-Berger B, Honda S (2015). Health-related quality of life among women in rural Bangladesh after surgical repair of obstetric fistula. Int J Gynecol Obstetr.

[24] Mselle LT, Evjen-Olsen B, Moland KM, Mvungi A, Kohi TW (2012). Hoping for a normal life again: reintegration after fistula repair in rural Tanzania. J Obstet Gynaecol Canada.

[25] Johnson KJ (2007). Incontinence in Malawi: analysis of a proxy measure of vaginal fistula in a national survey. Int J Gynecol Obstetr.

[26] Goh JT, Sloane KM, Krause HG, Browning A, Akhter SJ (2005). Mental health screening in women with genital tract fistulae. BJOG Int J Obstetr Gynaecol.

[27] Weston K, Mutiso S, Mwangi JW, Qureshi Z, Beard J, Venkat PJ (2011). Depression among women with obstetric fistula in Kenya. Int J Gynecol Obstetr.

[28] Senra C, Pereira MG (2015). Quality of life in women with urinary incontinence. Rev Assoc Méd Bras.

